# Developmental stage and lower quadriceps flexibilities and decreased gastrocnemius flexibilities are predictive risk factors for developing Osgood–Schlatter disease in adolescent male soccer players

**DOI:** 10.1007/s00167-023-07378-z

**Published:** 2023-03-31

**Authors:** Seira Takei, Suguru Torii, Shuji Taketomi, Satoshi Iizuka, Michio Tojima, Soichiro Iwanuma, Yukako Iida, Sakae Tanaka

**Affiliations:** 1 Department of Orthopaedic Surgery, Sensory and Motor System Medicine, Graduate School of Medicine, The University of Tokyo, Tokyo, Japan; 2 Waseda Institute of Human Growth and Development, Saitama, Japan; 3 Faculty of Sport Sciences, Waseda University, Saitama, Japan; 4 Japan Institute of Sport Sciences, Tokyo, Japan; 5 School of Health Sciences, Tokyo International University, Saitama, Japan; 6 Department of School Education, Teikyo University of Science, Tokyo, Japan; 7 Faculty of Sport Science, Surugadai University, Saitama, Japan

**Keywords:** Osgood–Schlatter disease, Knee, Peak height velocity age, Soccer, Quadriceps

## Abstract

**Purpose:**

This study aimed to elucidate the influential predictive risk factors of Osgood–Schlatter disease (OSD) on the support (non-kicking) leg among adolescent soccer players considering peak height velocity (PHV) age and investigate the cut-off values of the predictive variables.

**Methods:**

A cohort of 302 Japanese adolescent male soccer players aged 12–13 years were followed over 6 months. All players underwent physical examination, tibial tubercle ultrasonography, anthropometric and whole-body composition measurements, and muscle flexibility test of the support leg at the baseline. The developmental stage was evaluated from the PHV age. The OSD of the support leg was diagnosed 6 months later; players were divided into the OSD and control (CON) groups. The predictive risk factors were analyzed by multivariate logistic regression analysis.

**Results:**

There were 42 players who had developed OSD at baseline and they were excluded from the study. Among the 209 players, 43 and 166 belonged to the OSD and CON groups, respectively. The predictive risk factors of OSD development were PHV age ± 6 months at baseline (*p* = 0.046), apophyseal stage of tibial tuberosity maturity at baseline (*p* < 0.001), quadriceps flexibility ≥ 35° at baseline (*p* = 0.017), and decrease in gastrocnemius flexibility in 6 months (*p* = 0.009).

**Conclusion:**

PHV age ± 6 months at baseline, apophyseal stage of the tibial tuberosity at baseline, quadriceps flexibility ≥ 35° at baseline, and decrease in gastrocnemius flexibility in 6 months are predictive risk factors of OSD development in the support leg among adolescent male soccer players. It is crucial to know the PHV age of each player, and not only the flexibility of quadriceps muscle but also the gastrocnemius should be monitored to predict OSD.

**Level of evidence:**

II.

## Introduction

Osgood–Schlatter disease (OSD) is an apophysitis of the tibial tuberosity during the growth period caused by repetitive strain on the knee extensor mechanism. A peak in boys aged 10–15 years is well documented [4, 15]. The incidence of OSD in adolescent athletes is approximately 10–20% [16, 23] and is specifically high in athletes who participate in high-risk sports that require jumping, kicking, and running, such as soccer [3, 9, 26, 36]. Among soccer players, the support leg (non-kicking) tends to develop OSD than the kicking leg [9, 13, 47], since higher muscle activation of the quadriceps femoris muscle occurs on the support leg than on the kicking leg during the kicking motion [5, 19]. Moreover, the relationships between OSD onset on the support leg and characteristics of kicking motion were clarified in several studies [43]. However, no studies have investigated the risk factors of OSD focusing only on the support leg of soccer players.

Many studies have investigated the risk factors of OSD from various aspects. OSD develops when the tibial tuberosity maturity is at the apophyseal or epiphyseal stage [8, 26]. Commonly reported risk factor is reduced flexibility of the quadriceps femoris muscle during the growth spurt [9, 10, 24, 28, 47]; however, the cut-off value for quadriceps femoris flexibility during OSD onset has never been investigated.

Many studies have reported the high prevalence of sports injuries around the age of peak height velocity (PHV) among adolescent soccer players, especially 6 months before or after the PHV age [6, 31, 46]. The PHV age can be predicted by the individual height history, and it indicates the period of the peak height growth per year. As the growth peak of the muscle and bone mass are also related to the PHV age [20, 22, 32], significant changes in musculoskeletal structures during the growth spurt may be associated with the onset of sports injuries among adolescents. The OSD onset may also have a strong association with PHV age; however, no prospective study has investigated the risk factor of OSD considering the PHV age.

This prospective study aimed to qualify the cut-off value of quadriceps femoris flexibility or any other influential risk factors to predict OSD onset on the support leg in adolescent male soccer players and to investigate the association with PHV age. The hypothesis of this study is that OSD onset may have a strong association with PHV age, and that there are several independent risk factors to predict OSD other than reduced flexibility of the quadriceps femoris muscle.

## Materials and methods

The present study was approved by the Ethics Committee of University of Tokyo, Japan (2018079NI). All participants and their parents provided written informed consent before participating, and they were informed of their study data.

A cohort of Japanese adolescent male soccer players on the same soccer team in Tokyo, Japan was followed for over 6 months. The team plays in a town recreation league, and these participants attended regular soccer practice after school and on weekends (five times a week, 1.5–2 h per session). A total of 302 boys aged 12–13 years (height, 151.7 ± 7.4 cm; body weight, 41.4 ± 6.7 kg; body mass index [BMI], 17.9 ± 1.9 kg/m^2^) when they joined the soccer team between 2011 and 2018 were enrolled in this study. They had been playing in different teams before they began middle school, and the present study was conducted when they joined the same team from spring. The players were all in good health and free from disorders influencing growth.

### Measurements

Physical examination and classification of the tibial tuberosity maturation stage using ultrasonography were performed by an experienced orthopedic surgeon for all players at baseline (when they joined the club team in the spring of the first grade in middle school) and followed up 6 months later. Ultrasonography examination was conducted using SonoSite Edge II (Fujifilm, Tokyo, Japan) with 13-MHz (6–13 MHz) linear probes. The skeletal maturation stage of the tibial tuberosity was defined using the classification proposed by Ehrenborg [10] as cartilaginous, apophyseal, epiphyseal, or bony. Players who had tenderness and swelling of the tibial tuberosity and apophyseal or epiphyseal stage of tibial tuberosity maturity were diagnosed with OSD by an experienced orthopedic surgeon, as previously reported [8, 18, 33]. Height, body weight, sitting height, whole-body composition using dual-energy X-ray absorptiometry (DXA) scans, and muscle flexibility of the support leg (quadriceps femoris, hamstrings, and gastrocnemius) were measured at baseline and at 6 month follow-up. Height, body weight, and sitting height were measured to one decimal. The leg length was calculated by subtracting the sitting height from the body height. These measurements were performed on the same day. The individual height records in the past 6 years were collected to calculate the PHV age. Participants’ position in soccer was recorded by questionnaire.

### Body composition measurement

Body composition was measured using DXA scans (Delphi A-QDR pediatric whole-body scanner, version 12.4.3; Hologic Inc., MA, USA), and the lean mass of the trunk and lower extremities were evaluated. The lean mass derived by DXA has been used as an accurate estimation of skeletal muscle mass [30, 36]. Lean mass images were separated into discrete regions using anatomical landmarks that were visible in the scanned images. The trunk region was defined as the area from the first cervical vertebra to the femoral neck, which excluded the arm region from the axillary fossa to the end of the fingers, using the default DXA regional computer-generated lines on the anterior planogram view with manual adjustments, as described in the previous studies [12, 44]. The lower extremity region was defined as distal from the femoral neck.

### Muscle flexibility tests

**Fig. 1:**
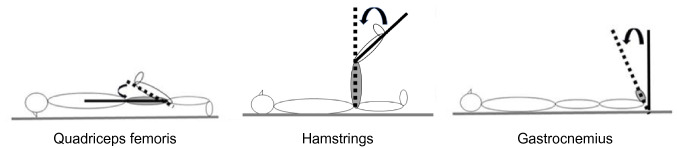
Measurement of the muscle flexibility of the support leg

Quadriceps flexibility was evaluated in degrees as knee flexion range of movement in the prone position using a standard goniometer. The hamstrings flexibility was evaluated by goniometric measurement of the angle of knee extension, with the hip angle kept at 90° of flexion. The gastrocnemius muscle flexibility was evaluated by the dorsiflexion angle of the ankle when maximally dorsiflexed in the supine position, with the knee extended (Fig. 1) The reliability is high for all of these measures (intraclass correlation coefficient of inter-rater reliability 0.86–0.99) [12, 14, 17, 22, 48], and the test–retest reliability was 0.94–0.95.

### Developmental stage

**Fig. 2:**
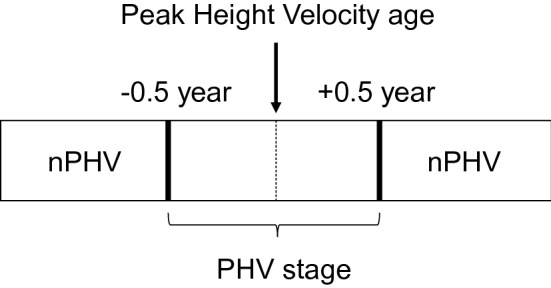
Definition of the PHV stage and nPHV stage

The PHV age of each participant, which is the age of peak height growth, was evaluated using the AUXAL 3.1 program (Scientific Software International, MD, USA) and their past height records from age 6 to 11 years and height measurements in this study. The developmental age of each participant was calculated by subtracting their chronological age from the PHV age. For example, if the developmental age was − 0.5 years, the participant would reach their PHV age in 0.5 years. Then, participants were classified into the PHV stage or nPHV stage according to their developmental age. When their developmental age ranges from − 0.5 years to + 0.5 years, they were classified into the PHV stage, and others were classified into the nPHV stage (Fig. 2).

**Fig. 3:**
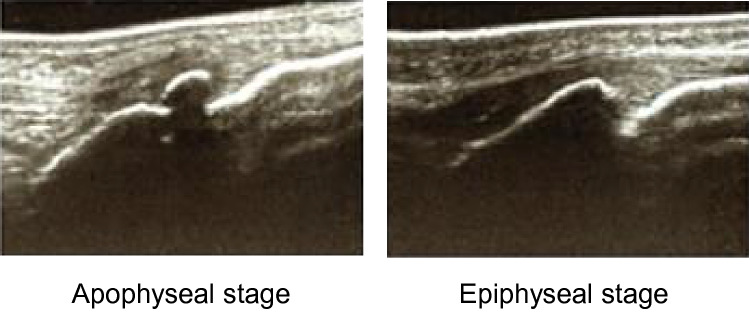
Skeletal maturation stage of the tibial tuberosity: apophyseal and epiphyseal stages

Players who were diagnosed with OSD at baseline were excluded from the analysis, and players whose tibial tuberosity maturity was at the apophyseal or epiphyseal stage (Fig. 3) at baseline were enrolled in the analysis. Participants who were diagnosed with OSD on the support leg 6 months from baseline were assigned to the OSD group. Players who did not develop OSD were assigned to the CON group. Players who developed OSD only on the kicking leg were excluded.

### Statistical analysis

According to a previous study [47], the estimated intragroup standard deviation of the quadriceps muscle was 8°, and the estimated difference between the OSD and CON group was > 5°. This study required more than 34 players with OSD to have 90% power and an alpha of 0.05.

Differences in the baseline anthropometric characteristics between the OSD and CON groups, such as the height, body weight, BMI, length of legs, lean mass of the support leg and trunk (adjusted by height), and differences in 6-month growth rate of these measurements, were assessed. The muscle flexibility of the support leg at baseline and the change in flexibility in 6 months were compared between the OSD and CON groups. The PHV age, soccer position, developmental stage (whether they are at the PHV stage or the nPHV stage), and tibial tuberosity maturity of the support leg at baseline (whether they are at the apophyseal or epiphyseal stage) were compared between the OSD and CON groups.

The results are reported as mean ± standard deviation (SD) and 95% confidence intervals (CI). Continuous variables that were normally distributed (Shapiro–Wilk test) were compared using the unpaired *t* test and those that failed the normality test were assessed with the Mann–Whitney *U* test to compare these two groups. Continuous variables with *p* values of < 0.05 were considered predictive variables, and the cut-off points for these variables were identified by drawing the receiver-operating curve (ROC) according to a previous report [2] and then divided into a high group and a low group. Categorized variables were compared using the Chi-squared test.

Then, all variables with *p* values of < 0.05 from the two-group comparisons were entered into a final multivariate logistic regression analysis to identify the most predictive variables of OSD. Odds ratios and 95% CIs from the final model were reported. The variance inflation factor (VIF) was calculated to evaluate the multicollinearity of the final model. All statistical analyses were performed using IBM SPSS Statistics for Windows, version 26.0 (IBM Corp., Armonk, NY, USA). Differences were considered significant for* p* values of < 0.05.

## Results

**Fig. 4:**
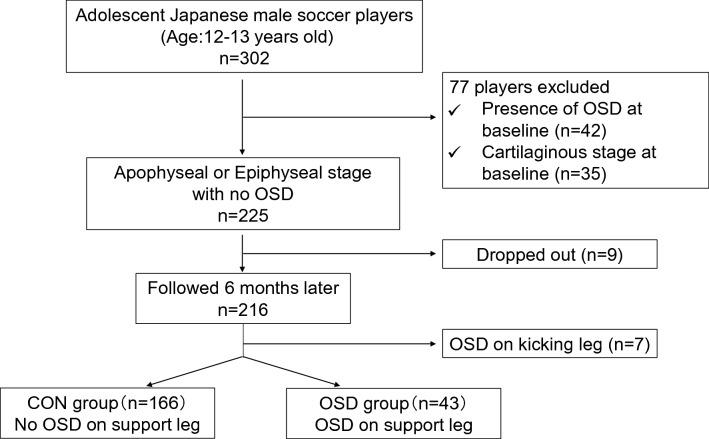
Flowchart of the exclusion criteria and dividing the participants into two groups

Two-hundred and sixteen players were followed up for 6 months from the baseline (Fig. 4). There were 43 players (19.9%) in the OSD group and 166 in the CON group at the follow-up assessments. In the OSD group, 18 players had OSD only on the support leg, and 25 players had bilateral OSD. Seven players with OSD only on the kicking leg and not on the support leg were excluded from the analysis.

No significant differences were found in anthropometric measurements, lean mass, and PHV age, and 6-month growth rate of these measurements (Table 1). The soccer position was not different between the two groups (Table 2).

**Table 1:** Comparison of anthropometric measurements at baseline and 6-month growth rate, and PHV age between the OSD and CON groups

	CON group (*n* = 166)	OSD group (*n* = 43)	*p* value
	Mean ± SD	Mean ± SD	
Baseline			
Height (cm)	152.8 ± 7.3	151.6 ± 6.6	n.s^a^
Body weight (kg)	42.5 ± 6.8	41.8 ± 5.9	n.s^a^
BMI (kg/m^2^)	18.1 ± 1.9	18.1 ± 1.6	n.s^a^
Leg length (cm)	71.7 ± 3.9	70.3 ± 3.6	n.s^a^
Lean mass of the support leg/height (kg/cm)	3.9 ± 0.5	3.7 ± 0.5	n.s^b^
Lean mass of the trunk/height (kg/cm)	10.3 ± 1.4	10.1 ± 1.0	n.s^a^
Peak height velocity age (year)	13.2 ± 1.0	13.2 ± 1.0	n.s^b^
Six-month growth rate			
Height (%)	2.5 ± 1.1	2.4 ± 1.5	n.s^a^
Body weight (%)	8.5 ± 11.3	6.7 ± 3.4	n.s^a^
BMI (%)	3.2 ± 11.0	0.9 ± 3.7	n.s^a^
Leg length (%)	2.5 ± 2.2	3.1 ± 1.8	n.s^a^
Lean mass of the support leg (%)	9.5 ± 4.7	8.9 ± 5.8	n.s^b^
Lean mass of the trunk (%)	10.3 ± 7.1	9.4 ± 5.2	n.s^a^

*BMI* body mass index, *PHV* peak height velocity, *SD* standard deviation

^a^Mann–Whitney *U* test

^b^Unpaired *t* test

**Table 2:** Comparison of soccer position between the OSD group and CON group

	CON group (*n* = 166)	OSD group (*n* = 43)	*p* value
Positions			
Goalkeeper (*n* = 23)	20 (87%)	20 (13%)	n.s
Defender (*n* = 64)	51 (79.7%)	13 (20.3%)	
Midfielder (*n* = 70)	57 (81.4%)	13 (18.6%)	
Forward (*n* = 40)	31 (77.5%)	9 (22.5%)	

*p* value obtained by the Chi-square test

The muscle flexibilities of the quadriceps femoris in the support leg at baseline were significantly different (lesser flexibility in the OSD group) between the two groups (*p* = 0.002), and the increase in gastrocnemius flexibility in the support leg 6 months later was smaller in the OSD group (*p* = 0.02) (Table 3). The optimal cut-off point for the quadriceps flexibility at baseline was ≥ 35° (AUC 0.65, 95% CI 0.556–0.743) and that for the increase in gastrocnemius flexibility in 6 months was < 0° (AUC 0.625, 95% CI 0.532–0.718), which was identified by drawing ROC.

**Table 3:** Comparison of the muscle flexibilities of the support leg at baseline and 6 months later between the OSD and CON groups

Muscle flexibility of the support leg (degree)	CON group (*n* = 166) Mean ± SD	OSD group (*n* = 43) Mean ± SD	*p* value
Baseline			
Quadriceps femoris	33.0 ± 7.3	41.3 ± 6.7	0.002^a^*
Hamstrings	39.7 ± 11.3	42.2 ± 4.5	n.s^b^
Gastrocnemius	9.1 ± 6.0	8.0 ± 5.9	n.s^a^
Changes in 6 months (degree)			
Quadriceps femoris	5.0 ± 9.2	2.1 ± 9.3	n.s^a^
Hamstrings	− 0.8 ± 13.3	0.4 ± 11.0	n.s^a^
Gastrocnemius	3.4 ± 8.2	0.5 ± 5.6	0.02^a^*

^a^Mann–Whitney *U* test

^b^Unpaired *t* test

^*^p < 0.05

**Fig. 5:**
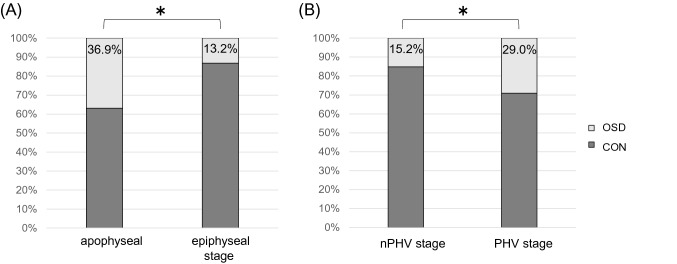
Tibial tuberosity maturity and developmental stage. **A** Comparing the rate of OSD development in the apophyseal stage and epiphyseal stage. **B** Comparing the rate of OSD development in the nPHV stage and PHV stage. *p < 0.05 obtained by the Chi-square test

The percentage in developing OSD was significantly higher in the apophyseal stage than in the epiphyseal stage (*p* < 0.001) at baseline and higher in the PHV group than in the nPHV group (*p* = 0.022) at baseline (Fig. 5).

Finally, four categorical variables were considered independent variables in the multivariate logistic regression analysis, including the PHV stage at baseline, apophyseal stage of tibial tuberosity maturity at baseline, quadriceps flexibility ≥ 35° at baseline, and decrease in gastrocnemius flexibility (< 0°) in 6 months. As a result, all four variables were identified as independent predictive risk factors of OSD development in the multivariate logistic regression analysis: PHV stage at baseline (odds ratio, 2.29), apophyseal stage of tibial tuberosity maturity at baseline (odds ratio, 4.56), quadriceps flexibility ≥ 35° at baseline (odds ratio, 2.73), and decrease in gastrocnemius flexibility in 6 months (odds ratio, 2.97) (Table 4).

**Table 4:** Multivariate logistic regression analysis results

Independent variables	Odds ratio	95% CI	*p* value
PHV stage (baseline)	2.29	1.01–5.18	0.046*
Apophyseal stage (baseline)	4.56	1.99–10.43	< 0.001*
Quadriceps femoris (baseline) ≥ 35°	2.73	1.19–6.24	0.017*
Gastrocnemius (changes in 6 months) < 0°	2.97	1.32–6.68	0.009*

*p* value obtained by logistic regression analysis

*p < 0.05

As the level of VIF of each variable was 1.0, no multicollinearity was noted in the final model.

## Discussion

The most crucial finding of this study was that the cut-off value of quadriceps and gastrocnemius muscle flexibility to predict OSD onset was clarified, and the strong association between PHV age and OSD onset was also demonstrated. In this longitudinal study, the most influential predictive risk factors of OSD development on the support leg in 6 months among adolescent male soccer players whose tibial tuberosity maturity was at the apophyseal or epiphyseal stage were investigated. The incidence of OSD was 19.9% during the 6-month period observation, which was similar to the reports of previous studies [7, 23, 32]. Four variables, PHV age ± 6 months at baseline, apophyseal stage of tibial tuberosity maturity at baseline, quadriceps flexibility ≥ 35° at baseline, and decrease in gastrocnemius flexibility (< 0°) in 6 months were identified as the most significant risk factors in the multivariate logistic regression model. The results of the present study may indicate the type of players who are more prone to developing OSD of the support leg and may add new information for creating an effective prevention program for OSD in soccer players.

### Muscle flexibility

In agreement with previous studies [9–11, 28, 32, 47], the decreased flexibility of the quadriceps was a risk factor for OSD development, and the quadriceps flexibility range of 35° was found to be the most appropriate cut-off point for prognostic screening of individuals at high risk of OSD development. This finding suggests that OSD prevention should focus on young athletes whose quadriceps flexibility is larger than 35°.

Multivariate logistic regression showed that decreased gastrocnemius flexibility (< 0°) in 6 months was also associated with a higher risk for OSD. Several studies [39, 47] have supported this result that decreased flexibility of the gastrocnemius is a risk factor of OSD. The reduced flexibility of the gastrocnemius on the support leg of soccer players may induce less ankle dorsiflexion at the support leg landing phase during the kicking motion. It may cause a deficit of the energy absorption at the ankle joint [2, 42] and also limit the body from moving forward [43], and therefore, knee joint torque may increase [1]. Focusing on the flexibility of the gastrocnemius not only the quadriceps is perhaps necessary to prevent OSD development in the support leg of soccer players.

No study has demonstrated a specific prevention program for OSD. Several studies have proved that muscle flexibilities can be improved by stretching [38, 40], but some studies not [25]. The efficacy of OSD-specific interventions focused on stretching quadriceps and gastrocnemius should be verified in the future.

### Tibial tuberosity maturity

In the present study, the apophyseal stage was significantly associated with OSD development compared with the epiphyseal stage. Previous cross-sectional study reported that the prevalence of OSD was the highest in the epiphyseal stage [51]; thus, many athletes may develop OSD at the apophyseal stage and maturate to the epiphyseal stage without healing. According to our study, we should especially pay attention to players in the apophyseal stage to prevent OSD.

### Developmental stage

To the best of our knowledge, this study is the first to reveal the developmental stage describing PHV age ± 6 months as a risk factor of OSD development prospectively. Consistent with our results, several studies have reported that the prevalence of sports injuries among adolescent soccer players is high during 6 months before or after the PHV age [6, 27, 46, 49], and van der Sluis et al. [45] demonstrated that coaches and trainers should be careful with the training and match load on the soccer players at around the PHV age to minimize the risk of injuries. Around the PHV age, not only the attachments of muscle tendons are stressed by the muscle shortening [41, 50], but the rapid growth of the limbs deteriorates balance and causes clumsy movements [21, 34, 35, 37]. Such changes in physical performance during the developmental stage of PHV age ± 6 months may also increase the mechanical stress on the immature stage of the tibial tuberosity.

This study has several limitations. First, this study was conducted in a specific population. The participants were boys, and they played soccer regularly on the same team. Second, the lean mass measured by DXA was determined as muscle mass in this study; however, muscle power was not evaluated. Third, the follow-up period was only 6 months. Since the current study was undertaken during a 6-month period, a certain number of players out of the 166 players in CON group will likely also develop OSD in the following 1–2 years. Following through the end of the athlete’s growth is necessary to understand the development of overuse injury during adolescence. Moreover, there were 42 players who had already developed OSD before 12 years old in this cohort. Thus, observing the players from younger ages is also needed. Fourth, the intensity of the sports training was not taken into account, which may become another crucial risk factor for OSD. Further study is needed to consider whether decrease in training intensity may be an effective prevention of OSD. Finally, this study investigated the risk factors of OSD by focusing only on the support leg of soccer players. As the biomechanical stress during kicking motion differs between the support leg and the kicking leg [5], this study focused on the support leg.

## Conclusion

This study demonstrated that PHV age ± 6 months at baseline, apophyseal stage of tibial tuberosity maturity at baseline, quadriceps flexibility ≥ 35° at baseline, and decreased gastrocnemius flexibility (< 0°) in 6 months are the most influential predictive risk factors for OSD onset on the support leg in 6 months among adolescent male soccer players. It is important to know the PHV age of each player, and the flexibility of gastrocnemius not only quadriceps muscle should be monitored to prevent OSD.

## Data Availability

The datasets analyzed during the current study are available from the corresponding author on reasonable request.

## Abbreviations

OSD: Osgood–Schlatter disease
PHV: Peak height velocity
BMI: Body mass index
DXA: Dual-energy X-ray absorptiometry
ROC: Receiver-operating curve
